# Gaucher Disease or Acid Sphingomyelinase Deficiency? The Importance of Differential Diagnosis

**DOI:** 10.3390/jcm13051487

**Published:** 2024-03-05

**Authors:** Miriam Giacomarra, Paolo Colomba, Daniele Francofonte, Marcomaria Zora, Giovanni Caocci, Daniela Diomede, Gaetano Giuffrida, Laura Fiori, Chiara Montanari, Annamaria Sapuppo, Anna Rita Scortechini, Nicola Vitturi, Giovanni Duro, Carmela Zizzo

**Affiliations:** 1 Institute for Biomedical Research and Innovation (IRIB), National Research Council (CNR), Via Ugo la Malfa 153, 90146 Palermo, Italy; miriam.giacomarra@irib.cnr.it (M.G.); paolo.colomba@irib.cnr.it (P.C.); daniele.francofonte@irib.cnr.it (D.F.); marcomaria.zora@irib.cnr.it (M.Z.); giovanni.duro@irib.cnr.it (G.D.); 2 Ematologia e Centro Trapianto di Midollo Osseo, Ospedale Businco, Via Jenner, 09124 Cagliari, Italy; giovanni.caocci@unica.it; 3 U.O.C. Ematologia e Trapianto, Ospedale “Mons. R. Dimiccoli”, Viale Ippocrate 15, 70051 Barletta, Italy; diomede.daniela@gmail.com; 4 Divisione Clinicizzata di Ematologia Sezione Trapianto di Midollo Osseo, Policlinico Vittorio Emanuele-Presidio Ospedaliero Ferrarotto, Via Citelli 6, 95124 Catania, Italy; gaegiuffrida@gmail.com; 5 Department of Pediatrics, Vittore Buzzi Children’s Hospital, University of Milan, Via Castevetro 32, 20154 Milan, Italy; laura.fiori@asst-fbf-sacco.it; 6 Department of Biomedical and Clinical Sciences, University of Milan, Via Giovanni Battista Grassi 74, 20157 Milan, Italy; chiara.montanari@asst-fbf-sacco.it; 7 Regional Referral Centre for Inborn Errors Metabolism, Pediatric Clinic, Department of Clinical and Experimental Medicine, University of Catania, Via S. Sofia 78, 95123 Catania, Italy; annamaria.pan@hotmail.it; 8 Azienda Ospedaliero Universitaria delle Marche, Clinica Ematologica, Via Conca 71, 60126 Ancona, Italy; annarita.scortechini@ospedaliriuniti.marche.it; 9 Department of Medicine-DIMED, Division of Metabolic Diseases, University Hospital, Via Giustiniani 2, 35128 Padova, Italy; nicola.vitturi@aopd.veneto.it

**Keywords:** Gaucher disease, acid sphingomyelinase deficiency, ASMD, glucocerebrosidase, acid sphingomyelinase, differential diagnosis

## Abstract

**Background:** Gaucher disease is a lysosomal storage disorder caused by functional glucocerebrosidase enzyme deficiency. Hepatosplenomegaly and hematological complications are found in both Gaucher disease and Acid Sphingomyelinase Deficiency, which is caused by acid sphingomyelinase dysfunction. The possible overlap in clinical presentation can cause diagnostic errors in differential diagnosis. For this reason, in patients with an initial clinical suspicion of Gaucher disease, we aimed to carry out a parallel screening of acid sphingomyelinase and glucocerebrosidase. **Methods:** Peripheral blood samples of 627 patients were collected, and enzymatic activity analysis was performed on both glucocerebrosidase and acid sphingomyelinase. The specific gene was studied in samples with null or reduced enzymatic activity. Specific molecular biomarkers helped to achieve the correct diagnosis. **Results:** In 98.7% of patients, normal values of glucocerebrosidase activity excluded Gaucher disease. In 8 of 627 patients (1.3%), the glucocerebrosidase enzymatic activity assay was below the normal range, so genetic GBA1 analysis confirmed the enzymatic defect. Three patients (0.5%) had normal glucocerebrosidase activity, so they were not affected by Gaucher disease, and showed decreased acid sphingomyelinase activity. SMPD1 gene mutations responsible for Acid Sphingomyelinase Deficiency were found. The levels of specific biomarkers found in these patients further strengthened the genetic data. **Conclusions:** Our results suggest that in the presence of typical signs and symptoms of Gaucher disease, Acid Sphingomyelinase Deficiency should be considered. For this reason, the presence of hepatosplenomegaly, thrombocytopenia, leukocytopenia, and anemia should alert clinicians to analyze both enzymes by a combined screening. Today, enzyme replacement therapy is available for the treatment of both pathologies; therefore, prompt diagnosis is essential for patients to start accurate treatment and to avoid diagnostic delay.

## 1. Introduction 

Gaucher disease (GD, OMIM #230800, ORPHA355) is a rare, autosomal, recessive genetic disorder determined by mutations in the GBA1 gene located on chromosome 1 (1q21), coding for the lysosomal enzyme, glucocerebrosidase (GCase, also called glucosylceramidase or acid β-glucosidase 1, EC: 4.2.1.25) [1,2,3,4,5,6,7]. In lysosomes, this enzyme hydrolyzes glucosylceramide (GlcCer) into ceramide and glucose; this impairment leads to the accumulation of GlcCer substrate in macrophages, also known as Gaucher cells. Penetrating the bone marrow, spleen, liver, and other organs, these cells are considered the main protagonists of the disease’s symptoms [1].

The phenotype is variable, and three clinical forms have been identified. Type 1 is the most common and causes no neurological damage: it is characterized by the association of hepatosplenomegaly (volume increase of the liver and spleen), skeletal pathology (pain, osteopenia, osteolytic lesions with fractures, bone infarcts, and osteonecrosis), and cytopenia (thrombocytosis, anemia, and, rarely, neutropenia).

Type 2 is characterized by neurological involvement, with onset before two years of life, a low psychomotor development, and a rapidly progressive course with death within two–four years of life. 

Type 3, although characterized by central nervous system involvement with infantile onset, has a slower progressive course and survival until the third or fourth decade of life [8,9].

A few of signs and symptoms of Gaucher disease are also found in Acid Sphingomyelinase Deficiency (ASMD) or Niemann Pick A/B disease [10]. It is caused by mutations in the SMPD1 gene coding for acid sphingomyelinase (ASM), which is necessary for sphingomyelin metabolism into ceramide and phosphocholine.

Since acid sphingomyelinase is essential for sphingolipid homeostasis, its failure causes reorganization of membrane lipid micro-domains, causing lipid abnormalities in the plasma membrane, lysosomes, and cellular signaling pathways [11]. 

The main signs and symptoms of ASMD affect the liver, spleen, heart, and respiratory, hematological, and skeletal systems. The presence or absence of neurological involvement, the extent of systemic disease, and the rate of disease progression make this systemic disorder highly clinically variable [12]. We can distinguish the following forms of ASMD: infantile neurovisceral ASMD, which is the most severe form, rapidly progressive, and uniformly fatal in early childhood [13]; chronic neurovisceral ASMD, which is more slowly progressive; and chronic visceral ASMD, which has onset of symptoms in childhood through adulthood and is associated with significant morbidity [14]. Patients with ASMD have clinical manifestations that overlap with other disorders: hepatosplenomegaly, hematological disorders, and bone alterations are also found in patients affected by Gaucher disease. In the case of chronic forms of ASMD, this overlap in symptoms and signs can lead to a long diagnostic odyssey for patients [12,15]. Moreover, Niemann Pick A/B is rarer than GD, with an estimated incidence between 1:100,000 and 1:264,000 in the general population [14]. Therefore, the presence of several clinical manifestations peculiar to both disorders should alert clinicians to think not only of Gaucher disease but also ASMD [10]. 

The clinical suspicion of both lysosomal disorders is confirmed by absent or deficient enzymatic analysis and supported by genetic investigation which must detect one causative mutation for each allele [16]. Moreover, molecular markers, such as, glucosphingosine (Lyso-Gb1) and chitotriosidase in Gaucher disease, and lysosphingomyelin (Lyso-SM) and lysosphingomyelin 509 (Lyso-SM-509), have also been found to be elevated in ASMD [17]. Several algorithms for pediatric metabolists and physicians involved in diagnosis and treatment of LSD have provided simple, convenient, and sensitive tools for diagnosing late-onset forms of GD and ASMDthat show the possible overlap in clinical presentation [10,16,18]. In this study, we present for the first time the results of the enzymatic activity of both glucocerebrosidase and acid sphingomyelinase in 627 patients with an initial clinical suspicion of Gaucher disease from hematology, internal medicine, and pediatric centers throughout Italy. 

In all of the patients, glucocerebrosidase enzyme activity was within the normal range except for eight samples, and clinical suspicion of Gaucher disease was confirmed by genetic analysis. At the same time, acid sphingomyelinase analysis carried out in all samples showed a normal activity except for three patients who had normal glucocerebrosidase enzyme activity, so they were not affected by Gaucher disease; in these patients, we found SMPD1 gene mutations responsible for ASMD. Biomarker levels further confirmed the genetic data.

## 2. Materials and Methods

A total of 627 samples of patients with an initial clinical suspicion of Gaucher disease from hematology, internal medicine, and pediatric centers throughout Italy were analyzed at the Center for Research and Diagnosis of Lysosomal Storage Disorders of IRIB-CNR in Palermo, Italy for the enzymatic activity values of glucocerebrosidase and acid sphingomyelinase. Enzymatic assays of both enzymes were carried out in all samples. Samples with GCase activity ≤ 2.5 nmol/h/mL (reference range: ≥2.5 nmol/h/mL) were analyzed for the GBA1 gene. The same diagnostic procedure was followed for acid sphingomyelinase: samples with values ≤ 1.7 nmol/h/mL (reference range: ≥1.7 nmol/h/mL) were analyzed for SMPD1. Samples carrying compound heterozygous or homozygous mutations in the GBA1 gene with a confirmed diagnosis of Gaucher disease were assessed for Lyso-GB1 values (normal range: ≤6.8 ng/mL) where possible. Samples with homozygous mutations in SMPD1 with a confirmed diagnosis of ASMD were assessed for lyso-SM509 values (normal range: ≤0.9 ng/mL) and for lyso_SM values (normal range: ≤46.3 ng/mL) where possible.

### 2.1. Patients

Peripheral blood samples were collected, using EDTA as an anticoagulant, and dried on specific absorbent paper (dried blood spot (DBS)). Genetic and enzymatic assays performed at the Center for Research and Diagnosis of Lysosomal Storage Disorders of CNR in Palermo were approved by the Hospital Ethics Committee of the University of Palermo; signed informed consent was obtained from all participants.

### 2.2. Glucocerebrosidase and Acid Sphingomyelinase Activity Assays

Glucocerebrosidase and acid sphingomyelinase analyses were performed using the Dried Blood Filter Paper test (DBFP) described by Chamoles et al. [19], with modifications. For the GCase enzymatic assay, the artificial substrate 4-methylumbelliferyl β-F-glucopyranoside (SIGMA-11.6 mmol/L in Phosphate-Citrate Buffer (CPB), pH 5.2), specific for GCase, conjugated with a fluorophore was used. The fluorescence values detected by the fluorometer (λex: 365 nm, λem: 448 nm) reflect the quantity of substrate degraded by the enzyme present in the analyzed sample. Fluorescence data were processed by an algorithm that provided for each sample an enzymatic activity value expressed in nmol/h/mL. Furthermore, we added to the reaction mixture a cell lysis solution at pH 5.2 (CPB, TRITON-X 100, SODIUM-TAUROCHOLATE) and CBE (Conduritol B epoxide), a specific inhibitor for the non-lysosomal β-glucosidase (NLGCase), to discriminate between GCase and NLGCase. Samples with the reaction mixture were incubated for 18 h at 37 °C in a thermomixer (900 RPM) covered with film. ASM activity assay was performed by CENTOGENE mass spectrometry. This test was performed for clinical purposes and its performance has been validated by CENTOGENE.

### 2.3. DNA Extraction 

DNA extraction was performed using EZ1 Advanced XL automatic extractor of QIAGEN S.r.l., Milano, Italy; the EZ1 Advanced XL DNA Investigator Card (a programmed card containing protocols for DNA extraction by filter paper) was used in combination with the EZ1 DNA Investigator kit of QIAGEN S.r.l.-Italy. Quantification of Genomic DNA required the use of the Eppendorf D30 biophotometer of Eppendorf—Hamburg, Germany.

### 2.4. GBA and SMPD1 Genetic Analyses 

The Long Polymerase Chain Reaction (Long PCR) method was applied after extensive study of the literature [20] to identify specific mutations of GBA1 and GBAP recombinants. 

Two pairs of PCR primers (Table 1) that exclusively amplify GBA1 and identify recombinant alleles with GBAP at 0.4 µM concentration in the reaction mixture were provided. Long PCR amplification of the two macroregions, exon 1–intron 5 and intron 5–exon 11 (two reaction mixtures for each sample), was performed using biotechrabbit™ Long-Range PCR Master Mix, 2X, following the basic protocol and cycling program in the reaction manual. PCR Enhancer and MgCl_2_ were not supplied in the mixture. The template DNA concentration was 50–100 ng in each reaction mixture. Exon 1–intron 5 and intron 5–exon 11 PCR products were purified by ExoSAP-IT™ PCR and used for sequencing the specific 1–5 exons and 6–11 exons of GBA1. Sequencing primers are listed in Table 1.

Six SMPD1 exons and their flanking intronic sequences were analyzed using five pairs of oligonucleotides (Table 1) at 0.4 µM concentration in the reaction mixture. Polymerase Chain Reaction (PCR) of 5 regions, exon 1–exon 6 (5 reaction mixtures for each sample), was performed using biotechrabbit™ Long-Range PCR Master Mix, 2X, following the basic protocol and cycling program in the reaction manual. PCR Enhancer and MgCl_2_ were not supplied in the mixture. The template DNA concentration was 40–60 ng in each reaction mixture. PCR products were purified and sequenced using the same PCR primers. Eurofins Genomics service performed Sanger sequencing. Sequence analysis was performed using LI-COR Align IR and Chromas bioinformatic programs 2.5. 

### 2.5. Biomarkers Assays

Detection of glucosylsphingosine (Lyso-Gb1), lysosphingomyelin-509 (Lyso SM-509) and lysosphingomyelin (Lyso SM) was performed in dried blood spots using the LC-MS/MS technique [21,22,23,24,25,26]. 

## 3. Results

We studied 627 subjects (average age: 56.3 years old, 58.5% males and 41.5% females, with average ages, respectively, of 55.4 and 57.5 years old) with clinical manifestations attributable to Gaucher disease. Of the 627 patients, 70.8% came from hematology centers, 17.1% from internal medicine, 6.8% from pediatrics, and 5.3% from other centers. In 619 patients (98.7%), normal activity values of glucocerebrosidase excluded Gaucher disease (Table 2). The average GCase activity in the unaffected patients was 8.3 nmol/h/mL. The average GCase activity in the 100 healthy control subjects was 8.7 nmol/h/mL. In 8 of the 627 patients (1.3%), deficient glucocerebrosidase activity was found and one homozygous mutation or two compound heterozygous mutations that confirmed the initial clinical suspicion of GD (Table 3). Homozygous or compound heterozygous mutations in the GBA1 gene were not found in the 100 healthy subjects studied. In 624 patients (99.5%), normal activity values of acid sphingomyelinase were found (Table 2). The average ASM activity in the unaffected patients was 10.1 nmol/h/mL. The average ASM activity in the 100 healthy control subjects was 10.6 nmol/h/mL. 

In 3 of the 627 subjects (0.5%) with an initial clinical suspicion of GD, we found normal glucocerebrosidase activity and acid sphingomyelinase activity reduction; homozygous mutations in the SMPD1 gene responsible for ASMD were found (Table 4). Homozygous or compound heterozygous mutations in the SMPD1 gene were not found in the 100 healthy subjects studied.

**Figure 1:**
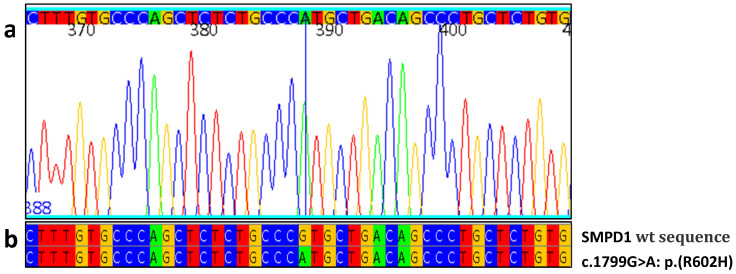
Index case mutation c.1799G>A. (**a**) Portion of the electropherogram of the SMPD1 exon 6 in index case; homozygous c.1799G>A is indicated by the blue line. (**b**) Portion of sequence of SMPD1 exon 6 in patient aligned with the corresponding sequence of a healthy control (wt). In the sequence and electropherogram, blue is for cytosine, red is for thymine, yellow is for guanine and green is for adenine.

Patient 1 was a 64-year-old man with moderate hepatosplenomegaly [27], thrombocytopenia, and a biopsy with Gaucher-like lysosomal accumulation. Normal glucocerebrosidase enzyme analysis values excluded the initial clinical suspicion of Gaucher disease. Nevertheless, the acid sphingomyelinase enzymatic assay result was 0.7 nmo/h/mL, below the normal range, and SMPD1 gene analysis detected homozygous c.1799G>A coding for p.R602H mutation (Figure 1) which is considered responsible for chronic visceral ASMD in homozygous or compound heterozygous mutations [28]. We also investigated Lyso-SM-509 accumulation, which was 4.7 ng/mL, above the normal range of ≤0.9 ng/mL.

**Figure 2:**
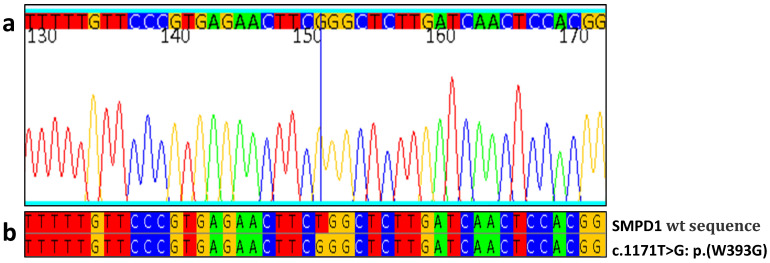
Index case mutation c.1171T>G. (**a**) Portion of the electropherogram of the SMPD1 exon 3 in index case; homozygous c.1171T>G is indicated by the blue line. (**b**) Portion of sequence of SMPD1 exon 3 in patient aligned with the corresponding sequence of a healthy control (wt). In the sequence and electropherogram, blue is for cytosine, red is for thymine, yellow is for guanine and green is for adenine.

Patient 11 was a 1-year-old boy with severe hepatomegaly (3.2 times normal volume) and severe splenomegaly [27], thrombocytopenia, anemia, and pulmonary involvement. On the basis of clinical signs, biochemical values, and radiological parameters, our first hypothesis was Gaucher disease with an intercurrent respiratory disease or acid sphingomyelinase deficiency (ASMD). Glucocerebrosidase enzyme analysis was within the normal range, while the acid sphingomyelinase enzymatic analysis result was below the normal range. SMPD1 genetic analysis revealed homozygous c.1171T>G coding for p.W393G mutation (Figure 2); according to the literature, this is considered responsible for chronic neurovisceral ASMD in homozygous or compound heterozygous mutations [29]. We also investigated Lyso-SM-509, which was 4.3 ng/mL, above the normal range of ≤0.9 ng/mL.

**Figure 3:**
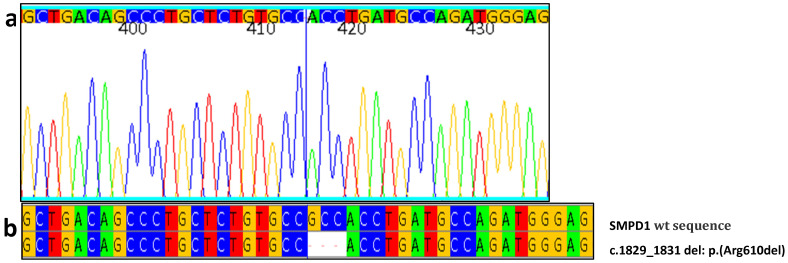
Index case mutation c.1829_1831del. (**a**) Portion of the electropherogram of the SMPD1 exon 6 in index case; homozygous c.1829_1831del is indicated by the blue line. (**b**) Portion of sequence of SMPD1 exon 3 in patient aligned with the corresponding sequence of a healthy control (wt). Dashes are missing nucleotides present in SMPD1 wt sequence. In the sequence and electropherogram, blue is for cytosine, red is for thymine, yellow is for guanine and green is for adenine.

Patient 210 was a 51-year-old female with anemia, severe hepatosplenomegaly [27], and recurrent ear infections. The main clinical suspicion of Gaucher disease was excluded by glucocerebrosidase enzymatic analysis, which was found within the normal range at 7.2 nmol/h/mL (normal range: ≥2.5 nmol/h/mL). The acid sphingomyelinase enzyme analysis values were very low, at 1.0 nmol/h/mL (normal range: ≥1.7 nmol/h/mL), which led us to carry out a genetic study of SMPD1, and homozygous deletion c.1829_1831del (p.Arg610del) (Figure 3) was detected. This mutation induces the elimination of one arginine amino acid in the protein, with a consequent change in the reading order. The mutation is considered responsible for chronic neurovisceral ASMD in homozygous or compound heterozygous mutations [30]. We also investigated Lyso-SM-509, which was 4.0 ng/mL, above the normal range of ≤0.9 ng/mL, and Lyso-SM, which was 148 ng/mL (normal range: ≤46.3 ng/mL).

## 4. Discussion

Gaucher disease is a rare enzymopathy with an incidence of 1/40,000 to 1/60,000 in the general population, but it can reach 1/800 births in the Ashkenazi Jewish population [31,32]; on the other hand, ASMD has an estimated incidence of ~1: 100,000 to date [33]. These two pathologies have several similar clinical features such as hepatosplenomegaly, thrombocytopenia, anemia, and pulmonary involvement, which is more serious in patients afflicted with ASMD [34]. Therefore, differential diagnosis should be part of common clinical practice in all patients with these signs and symptoms.

The inclusion of GD in differential diagnosis has been extensively implemented through education programs, diagnostic algorithms, and many studies that have evaluated the prevalence of GD in high-risk populations in different regions [16,35,36,37,38,39]. These programs, in addition to the development of specific and sensitive easy-to-use tools of great support to clinicians for the analysis of glucocerebrosidase activity on DBS, have contributed to the consideration of Gaucher disease as among the possible diagnostic hypotheses and to an increase in testing of suspected cases.

Unfortunately, in Acid Sphingomyelinase Deficiency, the problems leading to diagnostic delay are still observed today. Among the causes of this, the overlap in the clinical picture with other disorders such as Gaucher disease may be included. In the last years, several algorithms have been presented to help the scientific community in diagnosing GD and ASMD in subjects presenting with splenomegaly or hepatomegaly and further differentiating signs or symptoms [9,14].

Moreover, a recent study which collected data from 61 countries between 2017 and 2022 on samples analyzed for glucocerebrosidase and acid sphingomyelinase enzyme activities revealed that one in four cases suspected of Gaucher disease was a missed diagnosis of ASMD [40]. In our study, we analyzed both pathologies in 627 patients, with a diagnostic hypothesis of GD advanced by hematologists, internal medicine physicians, and pediatricians. In 8 of the 627 patients, glucocerebrosidase activity was reduced and one homozygous causative mutation or two biallelic causative mutations confirmed the initial clinical suspicion of Gaucher disease (Table 2 and Table 3). In 3 of the 627 patients with an initial suspicion of GD, glucocerebrosidase activity was normal; nevertheless, acid sphingomyelinase activity was decreased (Table 4). In each of these patients, SMPD1 homozygous mutations were found. Two patients had cDNA point mutations that caused amino acid changes, p.R602H and p.W393G, both responsible for Niemann Pick A/B [28,29]. The third patient had one deletion of three nucleotides (GCC) in position c.1829_1831 of the cDNA, which causes deletion of the amino acid arginine in position 610 of the protein (p.Arg610del), resulting in the reading code changing [30]. Lyso SM-509 was increased in all three patients, which helped us to confirm the Niemann Pick A/B diagnosis.

Our study, performed on patients with signs and symptoms strongly attributable to GD, confirmed the diagnosis of the disease only in 1.3% of patients. Because of the overlap in the clinical picture between Gaucher disease and ASMD, if we had analyzed only glucocerebrosidase activity we would have lost ~1 of every 200 patients afflicted with ASMD. Like all metabolic disorders, its complex diagnosis needs to be informed by medical cases, laboratory tests, and instrumental feedback [41]. For this reason, we believe that it is important to carry out differential clinical and molecular study of the two pathologies to avoid delaying the diagnosis.

Although some signs and symptoms, such as splenomegaly, hepatomegaly, and bone and blood involvement, are common to the two pathologies, others are less frequent or absent in Gaucher disease, such as pulmonary, hepatic, and cardiac involvement and dyslipidemia [12,34]. Therefore, their presence or absence should ring an alarm bell for clinicians, informing their suspicion of one or the other diagnosis. The combined screening of both enzymes could help clinicians in differential diagnosis.

Moreover, the development of the first specific therapy represents a step forward in the treatment of this rare disease that is associated with high morbidity and risk of premature death, as were other lysosomal diseases in the past [42,43,44].

## Figures and Tables

**Table 1:** PCR and sequencing primers for genetic analysis of GBA1 and SMPD1. Nucleotide sequences of the primers used to carry out the genetic study are shown.

GBA1 PCR Primers exon 1–intron 5	5′-3′ sequence
GBA_EX1FOR-PC	CTCCATGCAAATCTGTGTTC
GBA_EX5Rev-PC	GGCCTGAAAAAGCTAGAATG
GBA1 PCR Primers intron 5–exon 11	5′-3′ sequence
GBA_EX5FOR-PC	CCAGGATGATTGCGAACTC
GBA_EX11Rev-PC	TGCTGTGCCCTCTTTAGTC
GBA1 Sequencing Primers exon 1–intron 5	5′-3′ sequence
Ex1F seq GBA	AGATGAGAGGAAGCCAA
ex2R seq GBA	TGGTCTCAGTCACTCAAAAG
EX3F seq GBA	TCTTTTGAAACAGAGTCTT
EX4R_GBA	CAGAATGGGCAGAGTGAGAT
EX5F seq GBA	GGCCTCCCAAAGTGCTGG
GBA1 Sequencing Primers intron 5–exon 11	5′-3′ sequence
Ex6R seq bb	ATTGAGAGGCCCAAGGCT
Ex7R seq bb	CCCTAGAAAGGTTTCAAGCGA
EX8F_GBA	TCCAGGATCAGTTGCTCTTC
EX8R seq bb	AGTAAGAGGTCTGAGGTCTG
EX9F seq bb	TCTTACTAGTTTCACCAAAG
EX9R seq bb	AAGTTACGCACCCAATTGGG
EX9F seq bb2	CCTTGCCCTGAACCCCGAA
SMPD1 PCR Sequencing Primers	5′-3′ sequence
Ex1F SMPD1	TGAGCGCGGATTCTGACA
Ex1R SMPD1	AGCAAACTCAGTGATGGATT
Ex2F SMPD1	GTTGGCCTGGTTCCTCTGCT
Ex2R SMPD1	GTTCCCTTCTCCCTTCACTT
Ex3F SMPD1	CAGCACAGGAGGACCAGGA
Ex4R SMPD1	CAGCCTTCAGACACTCACCC
Ex5F SMPD1	CATCTCACCATCCCTGTTGT
Ex5R SMPD1	CTCCAACCTCCTTCCCCTAT
Ex6F SMPD1	TCCCTGGAGTTACCCTTGCT
Ex6R SMPD1	CTTGCCCTGCTTGCCTGGAA

**Table 2:** Characteristics of the subjects with initial clinical suspicion of GD. Characteristics of 627 subjects with clinical suspicion of Gaucher disease and the clinical centers from which they came. Enzyme activity is measured in nmol/h/mL; normal glucocerebrosidase values assayed in whole blood ≥ 2.5 nmol/h/mL. Normal acid sphingomyelinase values assayed in whole blood ≥ 1.7 nmol/h/mL. Normal Lyso-Gb1 values ≤ 6.8 ng/mL.

Overall	Subjects	Male	Female
Subjects (%)	627	367 (58.5)	260 (41.5)
Average age [min–max]	56.3 [1–90]	55.4 [1–90]	57.5 [1–86]
Hematology %	70.8	69.1	73.5
Internal Medicine %	17.1	17.4	16.5
Pediatrics %	6.8	7.6	5.8
Other %	5.3	6.0	4.2
GD patients (%)	8 (1.3)	5 (1.4)	3 (1.1)
Niemann Pick A/B patients (%)	3 (0.5)	2 (0.5)	1 (0.4)
Unaffected GD patients (%)	619 (98.7)	362 (98.6)	257 (98.8)
Unaffected ASMD patients (%)	624 (99.5)	365 (99.4)	259 (99.6)
Unaffected patients (%)	616 (98.2)	360 (98.1)	256 (98.5)
Average GCase activity in the probands	0.9	0.8	1.0
Average ASM activity in the probands	1.0	0.9	1.0
Average GCase activity in unaffected patients	8.3	8.6	7.9
Average ASM activity in unaffected patients	10.1	10.0	10.3

**Table 3:** Clinical and molecular data of patients with mutations in GBA1 gene. Normal glucocerebrosidase values assayed in whole blood ≥ 2.5 nmol/h/mL; normal acid sphingomyelinase values assayed in whole blood ≥ 1.7 nmol/h/mL; normal Lyso-Gb1 values ≤ 6.8 ng/mL; -- data not available; + present sign; - absent sign.

Patient No.	Gender/Age	ASM Activity	GCase Activity	Lyso-Gb1	GBA1 Mutations	Gaucher Disease	Splenomegaly	Thrombocytopenia	Anemia	Skeletal Pathology	Pulmonary Involvement	Other Signs
23	F/66	normal	0.4	--	G119R/N370S	Type I	+	+	-	-	-	-
82	M/53	normal	0.8	--	N370S/L444P	Type III	-	+	-	-	-	-
199	M/71	normal	0.3	346.0	R131C/N370S	Type I	+	+	-	-	-	-
206	M/23	normal	0.5	643.0	R131C/N370S	Type I	+	+	+	-	-	-
284	F/43	normal	1.7	332.0	N370S/L444P	Type I	+	+	--	-	-	-
464	M/77	normal	1.5	14.6	R131C/L444P	Type I	+	+	-	-	-	-
565	M/63	normal	1.0	82.0	N370S/N370S	Type I	+	+	--	--	--	--
627	F/12	normal	1.0	218.0	Q286R/N370S	Type I	+	+	+	+	-	Growth disturbance and leukopenia.

**Table 4:** Clinical and molecular data of patients with mutations in SMPD1 gene. Normal acid sphingomyelinase values assayed in whole blood ≥ 1.7 nmol/h/mL; normal glucocerebrosidase values assayed in whole blood ≥ 2.5 nmol/h/mL; normal Lyso-SM-509 values ≤ 0.9 ng/mL. Normal Lyso-SM values ≤ 46.3 ng/mL; -- data not available; + present sign; - absent sign.

Patient No	Gender/Age	GCase Activity	ASM Activity	Lyso-SM	Lyso-SM-509	SMPD1 Mutations	Splenomegaly	Piastrynopenia	Anemia	Skeletal Pathology	Pulmonary Involvement	Other Signs
1	M/64	8,8	0.7	--	4.7	R600H/R600H	+	+	-	-	-	-
11	M/1	16.7	1.2	--	4.3	W393G/W393G	+	+	+	-	+	-
210	F/51	7.2	1.0	148.0	4.0	c.1829_1831del/c.1829_1831del	+	+	+	-	-	Recurrent ear infections

## Data Availability

The original contributions presented in the study are included in the article, further inquiries can be directed to the corresponding author.
